# Beta-Hydroxybutyric Acid Inhibits Renal Tubular Reabsorption via the AKT/DAB2/Megalin Signalling Pathway

**DOI:** 10.1155/2022/3411123

**Published:** 2022-10-25

**Authors:** Minxia Zuo, Cheng Meng, Qian Song, Zhongai Gao, Xiao Cui, Jingyu Wang, Yongmei Li, Xiaochen Li, Chunyan Shan, Juhong Yang, Baocheng Chang

**Affiliations:** ^1^NHC Key Laboratory of Hormones and Development, Tianjin Key Laboratory of Metabolic Diseases, Chu Hsien-I Memorial Hospital, And Tianjin Institute of Endocrinology, Tianjin Medical University, Tianjin 300134, China; ^2^Wuhan Third Hospital, Tongren Hospital of Wuhan University, Wuhan 430070, China; ^3^Department of Respiratory and Infectious Diseases, Beijing You An Hospital, Capital Medical University, Beijing Institute of Hepatology, Beijing 100069, China

## Abstract

**Aim:**

Patients with diabetic ketosis often exhibit albuminuria. We previously found that acute hyperglycaemia can cause nephrotoxic injury. Here, we explored whether an excessive ketone body level causes kidney injury and the potential underlying mechanism.

**Methods:**

Fifty-six type 2 diabetes without ketosis (NDK group), 81 type 2 diabetes with ketosis (DK group), and 38 healthy controls (NC group) were enrolled. Clinical data were collected before and after controlling diabetic ketosis. Beta-hydroxybutyric acid (BOHB), an AKT activator, an AKT inhibitor, or plasmids encoding DAB2 were transformed into human renal proximal tubule epithelial cells (HK-2 cells).

**Results:**

The urinary albumin-to-creatinine ratio (ACR), transferrin (TF), immunoglobulin G (IgG), Beta2-microglobulin (*β*2-MG), retinol-binding protein (RBP), N-acetyl-beta-glucosaminidase (NAG), and Beta-galactosidase (GAL) were higher in the DK than NC and NDK groups. The proportion of patients with an increased urinary level of TF, IgG, *β*2-MG, RBP, NAG, or GAL was higher in the DK group too. After controlling ketosis, urinary microalbumin, TF, IgG, *β*2-MG, and RBP decreased significantly. In HK-2 cells, albumin endocytosis and megalin expression decreased with increasing BOHB concentration. Compared with BOHB treatment, BOHB with AKT activator significantly increased the DAB2, megalin levels and albumin endocytosis; the AKT inhibitor treatment exhibited the opposite effects. Compared with BOHB treatment, megalin expression and albumin endocytosis were significantly increased after BOHB with DAB2 overexpression treatment.

**Conclusions:**

Patients with diabetic ketosis may suffer from glomerular and tubular injuries that recover after ketosis control. High concentrations of BOHB downregulate megalin expression by inhibiting the AKT/DAB2/megalin signalling pathway and albumin endocytosis in proximal renal tubules.

## 1. Introduction

Diabetic ketoacidosis (DKA) is not confined to patients with type 1 diabetes [1, 2]; Swedish data suggest that patients with type 2 diabetes may account for one-third of all DKA cases [2]. Moreover, DKA caused by SGLT2 inhibitors (e.g., canagliflozin and dapagliflozin) is common [3, 4]. In clinical trials that enrolled 44,000 patients annually exposed to SGLT2 inhibitors, the rate of DKA development ranged from 0.16 to 0.76 events per 1,000 patient-years [5]. Whether DKA triggers further kidney damage is not clear. Approximately, 64.2% of DKA patients suffer from acute kidney injury (AKI) [6], and an increasing number of studies have reported that AKI accelerates the development of chronic kidney disease [7–9]. When kidney nephron regeneration was restored after AKI, the proximal tubules underwent pathological growth arrest, failed to differentiate, and became atrophic, rendering them unable to shrink [10] and thus increasing the risk of diabetic kidney disease.

Ketone bodies are metabolic fuel produced in the liver serving as energy sources for peripheral tissues. The principal ketone bodies are acetoacetate, 3-beta hydroxybutyric acid (BOHB), and acetone. Elevated levels of ketone bodies within specific ranges may be beneficial [11–14]. However, increased levels of circulating ketone bodies may damage the kidneys via an unknown mechanism [15].

Both albuminuria and acute hyperglycaemia are common in patients with newly diagnosed diabetes, especially those with DKA. We previously found that the urinary microalbumin level increased in a rat model of acute hyperglycaemia after high-glucose clamping. Using light and transmission electron microscopy, we found that acute hyperglycaemia damaged rat glomeruli and tubules to varying extents [16]. As patients with DKA may also evidence acute hyperglycaemia, there is a question of whether high blood ketone levels cause renal injury. To answer this question, we explored the differences in urinary indicators of renal injury between T2DM patients with and those without ketosis. We also evaluated kidney damage before and after ketosis control. We performed in vitro studies exploring how ketone bodies might cause renal injury.

## 2. Materials and Methods

### 2.1. Study Population

The diabetic patients were all outpatients from Tianjin Medical University Chu Hsien-I Memorial Hospital (2019.5-2019.6). They were divided into two groups according to urine ketone body: 56 people in the diabetic non-ketosis (NDK) group [urine ketone body test (-)], and 81 people in diabetic ketosis (DK) group [urinary ketone body test positive, (+) and above]. T2DM was defined according to American Diabetes Association's guidelines [17]. Thirty-eight healthy subjects from the Physical Examination Center of Tianjin Medical University Chu Hsien-I Memorial Hospital were included as the normal control (NC) group.

Inclusion criteria: (1) age between 18 and 60 years; (2) patients with type 2 diabetes. Exclusion criteria: (1) patients with previously diagnosed chronic kidney disease such as hypertensive nephropathy, chronic nephritis, diabetic nephropathy, and other diseases with abnormal liver function, patients with chronic hypoxia; (2) patients with lactic acidosis, hypertonic hyperglycaemia; (3) patients with urinary tract infection or other acute infections; (4) patients with stress state; (5) patients with severe cardiovascular and cerebrovascular diseases; (6) patients with tumors; (7) renal insufficiency (eGFR < 60 ml/min); (8) women during pregnancy or lactation; (9) patients with mental illness.

All patients analyzed in this study obtained written informed consent. This study follows the purpose of the Declaration of Helsinki and was reviewed and approved by the Medical Ethics Committee of Tianjin Medical University Chu Hsien-I Memorial Hospital (IRB number: DXBYYhMEC2019-16).

### 2.2. Collection and Analysis of Specimens

Blood specimens were collected from venous blood after fasting for more than 12 hours in all subjects. And then, they were centrifuged at 3000 r/min for 10 min at 4°C, taken the upper serum and packed in 1.5 ml EP tubes and then stored in a −80°C refrigerator. Morning 10 ml of random clean mid-section urine was collected for all subjects upon their first visit to our clinic. Urine specimens were centrifuged at 3000 r/min for 30 min at 4°C, taken the upper serum and packed in 2 ml EP tubes, and then stored in a −80°C refrigerator. After treatment of ketosis, urine negative for ketones were also collected in 29 patients in the DKA group. 7600A-020 Hitachi automatic biochemical analyzer was used to determine serum, liver and kidney functions, glycosylated hemoglobin, fasting blood glucose, blood lipids, and blood electrolytes. The estimated glomerular filtration rate (eGFR) was calculated using the EPI formula. The plasma osmotic pressure was calculated according to the formula: plasma osmotic pressure (POP) = 2 ([NA+] + [K+]) + blood sugar + urea nitrogen (mmol/l). An automatic urine analyzer was used to detect urine ketone bodies, and a urine biochemical detector (Roche, USA) was used to detect kidney injury indicators: UMA, uCr, urine TF, urine IgG, urine *β*2-MG, urine GAL, urine NAG, and urine RBP. ACR = UMA/uCr mg/g. ACR ≥ 30 mg/g was defined as (+), urine *β*2 − MG ≥ 0.3 mg/l was defined as (+), urine GAL ≥ 15 U/l was recorded as (+), urine IgG ≥ 17.5 mg/l was recorded as (+), urine NAG ≥ 12 U/l was recorded as (+), urine RBP ≥ 0.7 mg/l was recorded as (+), urine TF ≥ 5 mg/l was recorded as (+).ELISA kits (Hermes Criterion Biotechnology, Canada) were used to measure the blood level of BOHB.

### 2.3. Cell Culture

Human renal cortex proximal tubule epithelial cells (HK-2 cell line, purchased from the Chinese Type Culture Collection) were grown in DMEM/F12 medium (Hyclone, USA) and 10% fetal bovine serum (FBS; Gibico, USA) in humidified air (5% CO_2_) at 37°C. Then, cells were treated with the following agents: 0 mmol/l, 0.5 mmol/l, 1 mmol/l, 2 mmol/l, 3 mmol/l, and 4 mmol/l BOHB (BOHB, Sigma, USA) or 0 mmol/l, 0.5 mmol/l, 1 mmol/l, 2 mmol/l, 4 mmol/l, 8 mmol/l, and 16 mmol/l acetoacetic acid (AcAc, LinkChem, Shanghai, China) for 24 hours.

### 2.4. Western Blotting

The proteins were abstracted from cells or kidney cortex, separated using sodium dodecyl sulfate-polyacrylamide gel electrophoresis (SDS-PAGE), transferred to a polyvinylidene fluoride membrane (MilliporeSigma, Burlington, MA, USA), and then combined with incubate overnight with primary antibodies against megalin (1 : 1,000; Biorbyt), AKT (1 : 1,000; CST, USA), P-AKT (1 : 1,000; CST, USA), DAB2 (1 : 1,000; Proteintech, China), or *β*-actin (1 : 1,000; Abclonal, Wuhan, China). The membrane was then reacted with the secondary antibody. The chemiluminescence signal was recognized by ECL reagent (Advansta, CA, USA). Blots were quantified using ImageJ software.

### 2.5. Measurements of the Fluorescence Intensity of the Endocytosis of FITC-BSA in HK-2 Cells

After stimulation with different concentrations of BOHB (Sigma, USA) for 24 hours, HK-2 cells were treated with 500 ug/ml fluorescein isothiocyanate-labeled bovine serum albumin (FITC-BSA, Solarbio, Beijing, China) for 2 hours in a dark environment. The cells were washed twice with 0.01 mol/l PBS, and the fluorescence intensity released from the cells was measured with a microplate reader (Biotek, Germany) at an excitation wavelength of 493 nm and an emission wavelength of 550 nm.

### 2.6. RNA Extraction and Quantification of Gene Expression

EZNA® Total RNA Kit (Omega, GA, USA) was used to extract total RNA from the cells. cDNA was synthesized using the reverse transcription system kit (Thermo, USA). Real-time qPCR was performed using the SYBR Green PCR kit (Sangon Biotech, Shanghai, China).

### 2.7. Plasmid Transfection

DAB2 gene overexpressed clone (Tianjin In Vitro Biotechnology Co., Ltd.) and homologous negative control were purchased from GenePharma (Shanghai, China). According to the manufacturer's instructions, lipofectamine 2000 reagent (Invitrogen, CA, USA) was transfected into cells at 50–100 mmol/l.

### 2.8. Statistical Analyses

SPSS22.0 (SPSS, Chicago, IL, USA) statistical software was used for statistical analysis. Quantitative data with a normal distribution are expressed as the mean ± standard deviation (SD). Quantitative data with a nonnormal distribution are expressed as the median (first quartile, third quartile). One-factor analysis of variance was used to analyze differences between groups for data with a normal distribution, whereas nonparametric tests were used to analyze data with nonnormal distribution. A value of *P* < 0.05 was considered statistically significant.

## 3. Results

### 3.1. General Data

The diabetic course and the levels of fasting plasma glucose, HbA1c, albumin, total cholesterol, triglycerides, low-density lipoprotein-cholesterol, potassium, blood urea nitrogen, estimated glomerular filtration rate, and serum uric acid differed significantly among the NC group, NDK group, and DK group (*P* < 0.05). However, age, sex ratio, liver function, serum creatinine level, and the levels of high-density lipoprotein-cholesterol, sodium, and plasma osmotic pressure did not differ significantly among the three groups (all *P* > 0.05) (Table 1).

### 3.2. Glomerular and Tubular Injuries

#### 3.2.1. Injuries in DK Patients

**Figure 1:**
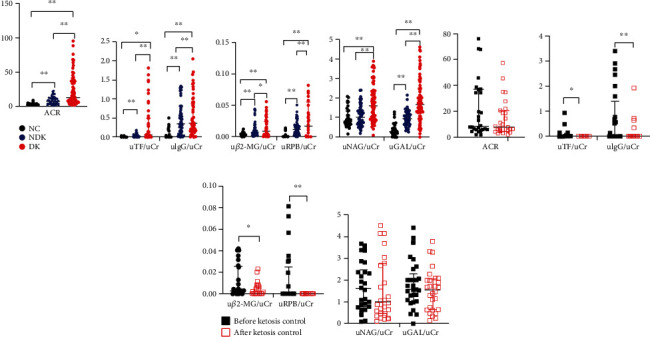
Glomerular and tubular injury in NC, NDK, and DK patients. Quantitative analysis of ACR (a), uTF/uCr and uIgG/uCr (b), u*β*2-MG/uCr and uRBP/uCr (c), uNAG/uCr and uGAL /uCr (d) in three groups , ACR (e), uTF/uCr and uIgG/uCr (f), u*β*2-MG/uCr and uRBP/uCr (g)and uNAG/uCr and uGAL /uCr (h) after control of diabetic ketosis. ACR, albumin creatinine ratio;u*β*2-MG, urine *β*2-microglobulin; uGAL, urine *β*-galactosidase; uIgG, urine Immunoglobulin G; uNAG, urine N-acetyl-*β*-glucosaminidase; uRBP, urine retinol-binding protein; uTF, urine Transferrin; uCr, urinary creatinine; NC, normal control; NDK, non-diabetic ketosis; DK, diabetic ketosis. ^∗∗^Represents P < 0.01; ^∗^ represents P < 0.05.

All kidney injury indicators were normalised to the urinary creatinine levels. The levels of glomerular injury indicators (ACR, urinary TF, and IgG) and renal tubular injury markers (urinary *β*2-MG, RBP, and GAL) tended to increase in the order of the NC, NDK, and DK groups, with statistically significant differences among the three groups (all *P* < 0.05) (Figures 1(a)–1(d)). The urinary NAG level was significantly higher in the DK group compared with the NC and NDK groups (*P* < 0.05) but was not different between the NC and NDK groups (Figure 1(d)).

#### 3.2.2. Rates of Glomerular and Tubular Injuries

**Figure 2:**
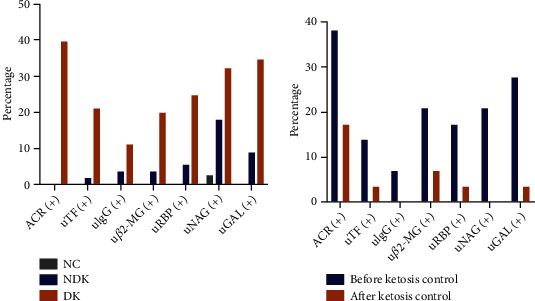
Percentages of patients with increased glomerular and tubular injury in DK patients. ACR, albumin creatinine ratio; u*β*2-MG, urine *β*2-microglobulin; uGAL, urine *β*-galactosidase; uIgG, urine Immunoglobulin G; uTF, urine Transferrin; uNAG, urine N-acetyl-*β*-glucosaminidase; uRBP, urine retinol-binding protein; NC, normal control; NDK, non-diabetic ketosis; DK, diabetic ketosis. ACR≥30mg/g was defined as (+), c2 = 45.446, p < 0.001 between NC,NDK, and DK groups, c2 = 3.107, p = 0.07 between before and after ketosis control; urine *β*2-MG≥0.3mg/l was defined as (+), c2 = 14.960, p = 0.001 between NC, NDK, and DK groups, c2 = 2.320, p = 0.128 compared before and after ketosis control; urine GAL≥15U/l was recorded as (+), c2 = 25.505, p < 0.001, between NC, NDK, and DK groups, c2 = 6.444, p = 0.01 between before and after ketosis control; urine IgG≥17.5mg/l was recorded as (+), c2 = 6.451, p = 0.04 between NC,NDK and DK groups, c2 = 2.071, p = 0.15 between before and after ketosis control; urine NAG≥12U/l was recorded as (+), c2 = 14.004, p = 0.001 between NC,NDK and DK groups, c2 = 6.692, p = 0.01 between before and after ketosis control; urine RBP≥0.7mg/l was recorded as (+), c2 = 18.187, p < 0.001 between NC, NDK, and DK groups, c2 = 2.974, p = 0.08 between before and after ketosis control; urine TF≥5mg/l was recorded as (+), c2 = 18.975, p < 0.001 between NC,NDK and DK groups, c2 = 1.970, p = 0.16 between before and after ketosis control.

Compared with the NC and NDK groups, the rate of an increased ACR was significantly higher in the DK group (*P* < 0.05) (Figure 2(a)). Compared with the NC group, the rates of increased urinary TF, IgG, *β*2-MG, RBP, NAG, or GAL levels were higher in the NDK and DK groups, and the DK group exhibited the highest rates of glomerular and tubular injuries (all *P* < 0.05) (Figure 2(a)).

### 3.3. Glomerular and Tubular Injuries before and after Ketosis Control

#### 3.3.1. Glomerular and Tubular Injury Indicators

To assess the impact of ketosis on renal function, we observed changes in the levels of biomarkers of glomerular and tubular damage after ketosis was controlled. We found no significant difference in the fasting plasma glucose level before and after ketosis control (*P* > 0.05) (Table 2). Compared with before ketosis control, the urinary levels of ketones, TF, and IgG decreased significantly after control (all *P* < 0.05) (Table 2 and Figure 1(f)); there was a decreasing trend in the ACR (*P* > 0.05) (Figure 1(e)). Renal tubular injury indicators (urinary *β*2-MG and RBP levels) decreased significantly after controlling ketosis (both *P* < 0.05) (Figure 1(g)), but the levels of urinary NAG and GAL did not change significantly (both *P* > 0.05) (Figure 1(h)).

#### 3.3.2. Rates of Kidney Injuries after Ketosis Control

Compared with before controlling ketosis, the proportions of patients with increased urinary NAG and GAL levels decreased significantly after ketosis control (both *P* < 0.05) (Figure 2(b)), but no significant differences were found in the proportions of patients with increased urinary levels of ACR, TF, IgG, *β*2-MG, or RBP (all *P* > 0.05).

### 3.4. Effects of BOHB and Acetoacetate on Endocytosis and Megalin Expression in HK-2 Cells

**Figure 3:**
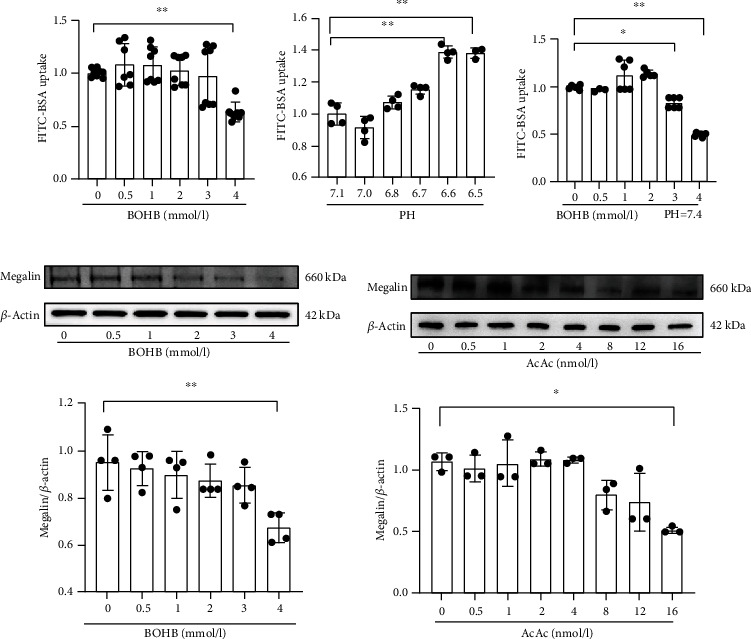
Effects of different concentrations of BOHB and acetoacetate on endocytosis and megalin expression of HK-2 cells. (a) Quantification of FITC-BSA uptake in HK2 cells in different concentrations of BOHB. (b) Quantification of FITC-BSA uptake in HK2 cells in the indicated pH level. pH 7.1 represents 0 mmol/l BOHB; pH 7.0 represents 0.5 mmol/l BOHB; pH 6.8 represents 1 mmol/l BOHB; pH 6.7 represents 2 mmol/l BOHB; pH 6.6 represents 3 mmol/l BOHB; pH 6.5 represents 4 mmol/l BOHB. (c) Quantification of FITC-BSA uptake in HK2 cells in different concentrations of BOHB when pH was adjusted to 7.4. (d) Representative western blotting images and quantification of megalin in different concentrations of BOHB. (E) Representative western blotting images and quantification of megalin in different concentrations of AcAc. AcAc, acetoacetate; BOHB, beta-hydroxybutyric acid. Data are expressed as mean ± SD. ^∗∗^Represents P < 0.01; ^∗^represents P < 0.05.

To further evaluate the effects of ketone bodies on renal tubular function, we treated HK-2 cells with BOHB and acetoacetate. As the BOHB level increased, albumin endocytosis in HK-2 cells decreased; the decrease was maximum at a BOHB concentration of 4 mmol/l (*P* < 0.05) (Figure 3(a)). To exclude any effect of pH, we established a pH control group for each BOHB concentration. As the pH decreased, albumin endocytosis increased and significantly so (compared with control cells) when the pH reached 6.6 (*P* < 0.05) (Figure 3(b)). As the pH of human fluids is 7.35–7.45 under physiological conditions, we adjusted the pH in all BOHB treatment groups to 7.4, after which albumin endocytosis decreased (Figure 3(c)). Megalin expression was minimal at a BOHB concentration of 4 mmol/l (*P* < 0.05) (Figure 3(d)) or an acetoacetate concentration of 16 mmol/l (*P* < 0.05) (Figure 3(e)).

### 3.5. BOHB Inhibits Endocytosis in Proximal Tubule Epithelial Cells via the AKT/DAB2/Megalin Signalling Pathway

#### 3.5.1. Effects of Different Concentrations of BOHB on AKT/DAB2 Expression

**Figure 4:**
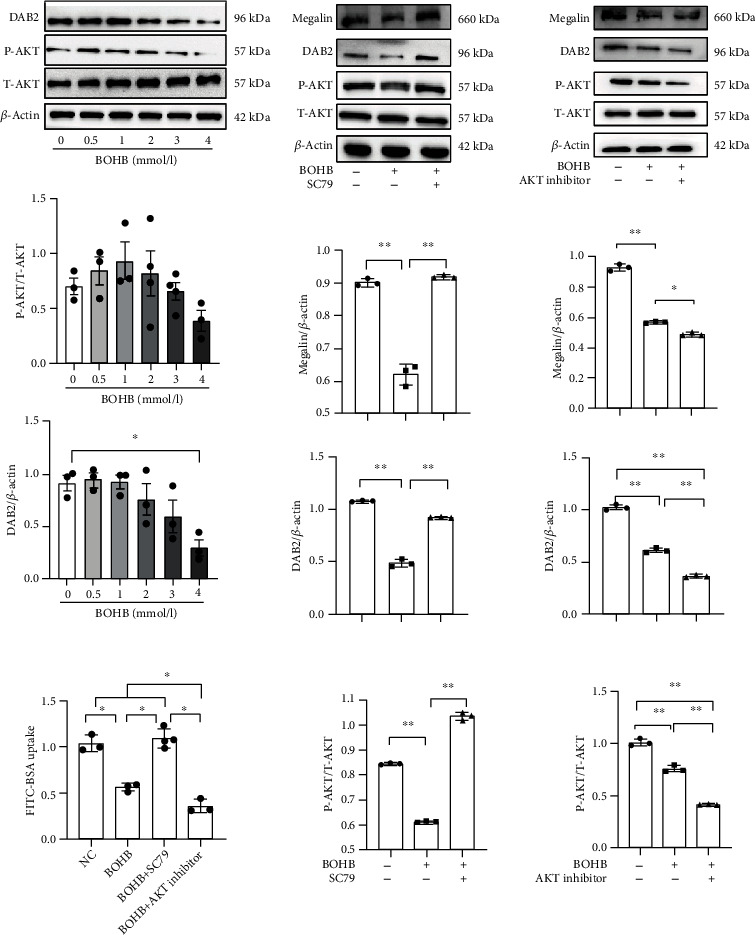
Beta-hydroxybutyrate affects endocytosis of proximal tubule epithelial cells through AKT/DAB2/megalin signaling pathway. (a) DAB2, P-AKT, and T-AKT in indicated concentrations of BOHB. (b) Megalin, DAB2, P-AKT, and T-AKT in indicated concentrations of BOHB with SC79 or not. (c) Megalin, DAB2, P-AKT, and T-AKT in indicated concentrations of BOHB with AKT inhibitor or not. (d) FITC-BSA uptake level in HK2 cells. BOHB, beta-hydroxybutyric acid; NC, normal control; SC79, AKT activator. Data are expressed as mean ± SD. ^∗∗^Represents P < 0.01; ^∗^represents P < 0.05.

We found that as the BOHB concentration increased, phosphorylation of AKT (P-AKT/T-AKT) decreased, although statistical significance was not attained. DAB2 expression also gradually decreased, with a significant difference observed from 4 mM BOHB (*P* < 0.05) (Figure 4(a)).

#### 3.5.2. Effects of the AKT Activator SC79 on AKT, DAB2, and Megalin Expression

SC79 is a selective AKT activator. Compared with the NC group, BOHB reduced the levels of P-AKT/T-AKT, DAB2, and megalin (all *P* < 0.05). However, SC79 rescued all of these decreases (all *P* < 0.05) (Figure 4(b)).

#### 3.5.3. Effects of an AKT Inhibitor on AKT, DAB2, and Megalin Expression

AKT inhibitors selectively inhibit AKT1/AKT2 activities. Compared with the NC group, BOHB reduced the levels of P-AKT/T-AKT, DAB2, and megalin. After addition of the AKT inhibitor, the levels of P-AKT/T-AKT, DAB2, and megalin further decreased (Figure 4(c)).

#### 3.5.4. Effects of an AKT Inhibitor and Activator on Endocytosis in HK-2 Cells

Compared with the NC group, BOHB reduced albumin endocytosis in HK-2 cells (*P* < 0.05). Addition of SC79 increased endocytosis, while addition of AKT inhibitor further decreased endocytosis (*P* < 0.05) (Figure 4(d)).

#### 3.5.5. Effect of DAB2 Overexpression on Megalin Expression in HK-2 Cells Treated with BOHB

**Figure 5:**
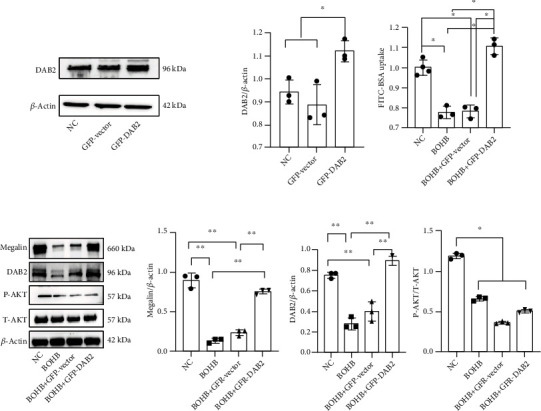
Effects of DAB2 overexpression on AKT/DAB2/megalin expression. (a) DAB2 level in HK-2 cells transfected with GFP-Vector or GFP-DAB2. (b) FITC-BSA uptake levels in HK-2 cells transfected with GFP-Vector or GFP-DAB2. (c) Megalin, DAB2, and AKT in HK-2 cells transfected with GFP-Vector or GFP-DAB2. BOHB, beta-hydroxybutyric acid; NC, normal control; GFP-DAB2, plasmid encoding DAB2; GFP-Vector, plasmid encoding control. Data are expressed as mean ± SD. ^∗∗^Represents P < 0.01; ^∗^represents P < 0.05.

Compared with the NC and green fluorescent protein (GFP)—vector groups, the DAB2 level was significantly increased in the GFP-DAB2 group (*P* < 0.05) (Figure 5(a)). The DAB2 and megalin levels were higher after BOHB with GFP-DAB2 treatment than after BOHB treatment or BOHB with GFP-vector treatment (*P* < 0.05) (Figure 5(c)).

#### 3.5.6. Effect of DAB2 Overexpression on Endocytosis in HK-2 Cells

We found no significant difference in albumin endocytosis between the BOHB with GFP-Vector and the BOHB treatments (*P* > 0.05). Compared with BOHB treatment, BOHB plus GFP-DAB2 treatment significantly increased endocytosis in HK-2 cells (*P* < 0.05) (Figure 5(b)).

## 4. Discussion

We found that type 2 diabetic patients with ketosis exhibited proteinuria. Compared with normal individuals and diabetic patients without ketosis, patients with ketosis evidenced increased levels of urinary markers of glomerular and renal tubular injury. After controlling the ketosis, the levels of these biomarkers (ACR, TF, IgG, RBP, and *β*2-MG) were reduced, indicating that glomerular and tubular injuries were partially repaired after ketosis control. In vitro, we found that BOHB impaired endocytosis by proximal renal tubule cells via the AKT/DAB2/megalin signalling pathway, which may explain how BOHB induces renal tubular injury.

We previously reported that acute or temporary hyperglycaemia caused glomerular and tubular damage [16]. Here, we found that ketosis triggered renal injury in a hyperglycaemia-independent manner. TF is a marker of glomerular injury, and it readily crosses the glomerular barrier because of its low molecular weight and low ion load [18]. Increases in urinary IgG and TF levels reflect high glomerular fluid pressure [18]. The urinary IgG level rises prior to the development of microalbuminuria, accompanied by an increase in the urinary TF level [19]. The levels of glomerular and renal tubular injury markers were increased significantly in diabetic kidney disease patients [20, 21]. We found that ketosis may play an important role in renal injury. Compared with the NDK group, the urinary IgG and TF levels were significantly increased in the DK group, indicating that ketone bodies promote glomerular injury. The estimated glomerular filtration rate was significantly higher in the DK group than NC and NDK groups. We speculate that hyperfiltration in patients with ketosis increases the glomerular fluid pressure, thus increasing the urinary excretion of IgG and TF.

The urinary *β*2-MG level is a sensitive and reliable indicator of renal tubular injury [21], and the urinary RBP level reflects the functional status of the proximal renal tubules [22]. Compared with the NDK group, the urinary levels of *β*2-MG and RBP were higher in the DK group. Thus, compared with diabetic patients without ketosis, those with ketosis have a higher rate of renal tubular injury. The urinary GAL and NAG levels are classical biomarkers of renal tubulointerstitial injury [23], with a high sensitivity for detecting structural injury to the renal tubules. Abnormal changes caused by ketosis have been rarely reported. We found that compared with the NDK group, the levels of urinary NAG and GAL were significantly higher in the DK group, indicating that ketosis plays a significant role in damage to renal tubular structures. Our study suggests that ketosis may cause renal tubular injuries, and these damages may be independent of hyperglycaemia.

Furthermore, we found that after ketosis control, although the blood glucose level did not significantly decrease, the urinary levels of TF, IgG, *β*2-MG, and RBP decreased dramatically, and those of ACR, TF, and IgG tended to decrease, suggesting that the glomerular and renal tubular injuries had been repaired to some extent. Early ketosis treatment is important to prevent kidney injury independent of that caused by hyperglycaemia; ketosis-related kidney injury is reversible in the short term.

Impaired renal tubular reabsorption triggers microalbuminuria (albumin leakage from glomeruli) [24–26]. The severity of proximal renal tubular injury determines the rate of chronic kidney disease progression [27, 28]. However, the mechanism of renal-tubular-related proteinuria is poorly understood. Proximal tubular reabsorption is controlled by megalin and disabled-2 (DAB2). Megalin is a low-density lipoprotein receptor expressed principally on the apical surfaces of proximal tubule epithelial cells. The megalin NPXY-like motif VENQNY plays a role in protein internalisation during endocytosis [29]. DAB2 is a cytoplasmic adaptor protein abundant in renal proximal tubules. DAB2 binds to the two NPXY motifs of megalin, and is essential for receptor protein-mediated endocytosis [30, 31]. In renal proximal tubule epithelial cells, DAB2 participates in megalin-mediated endocytosis by directly interacting with the megalin cytoplasmic tail during endocytosis [32], promoting protein absorption.

Dysregulated AKT signalling is associated with various human diseases [33, 34]. PKB/AKT expression directly affects DAB2 [35]. Megalin expression is decreased in DAB2^−/−^ mice [36], suggesting that DAB2 may participate in megalin regulation. However, the relevance of such observations in terms of how ketosis affects renal tubular reabsorption remains to be established. We found that a high concentration of BOHB inhibited megalin expression via the AKT/DAB2/megalin signalling pathway, in turn triggering endocytosis dysfunction of renal tubular cells. This may be why excess ketone bodies impaired renal reabsorption.

Ketogenic diets are increasingly used to promote weight loss and enhance lipid and glucose metabolism [37]. However, such diets are high in fat and low in carbohydrates; this may increase insulin resistance, promote abnormal glycolipid metabolism, aggravate vascular endothelial injury, and increase cardiovascular disease risk. Ketogenic diets may activate the renin–angiotensin–aldosterone system; reduce autophagy, and further increase hypertension, in turn triggering severe renal fibrosis, a decreased GFR, and creatinine accumulation [38]. Our work shows that excessive ketone bodies promote renal injury, although the damage was reversible in the short term. However, whether the damage is reversible in a longer term is unknown. Although we found no differences in blood glucose levels after ketosis control, it remains unclear whether other changes in body fluids or insulin levels restored renal function.

In summary, we found that in patients with diabetic ketosis, in addition to hyperglycaemia, ketone bodies can cause further injury to the kidney, as revealed by increased levels of urinary glomerular and tubular injury markers. Reducing the level of ketone bodies repaired renal injury. A high concentration of BOHB downregulated megalin expression and inhibited endocytosis in proximal tubule epithelial cells by regulating the AKT/DAB2/megalin signalling pathway.

## Figures and Tables

**Table 1:** General information in DK patients.

	NC	NDK	DK	*P* value
*N*	38	56	81	—
Age	44.61 ± 15.41	50.33 ± 10.76	51.41 ± 13.87	0.18
Gender (male/female)	21/17	31/25	51/32	0.11
Course (years)	0.00 (0,0)	9.00 (4,16)^a^	5.00 (0,7)^ab^	<0.01
FPG (mmol/l)	5.16 ± 0.41	9.51 ± 3.07^a^	10.49 ± 3.73^ab^	<0.01
HbA1c (%)	5.03 ± 0.48	8.22 ± 1.76^a^	9.58 ± 2.13^ab^	<0.01
TP (g/l)	73.81 ± 5.85	73.44 ± 4.99	72.47 ± 6.65	0.48
ALB (g/l)	45.84 ± 3.86	45.74 ± 3.91	43.53 ± 4.55^ab^	0.04
ALT (IU/l)	28.53 ± 19.84	27.40 ± 16.28	23.86 ± 13.98	0.29
AST (IU/l)	20.29 ± 7.28	23.77 ± 14.20	19.70 ± 9.99	0.12
TBIL (*μ*mol/l)	14.21 ± 5.26	14.04 ± 4.15	14.74 ± 6.96	0.77
IBIL (*μ*mol/l)	10.50 ± 4.45	10.46 ± 4.54	11.89 ± 9.91	0.45
DBIL (*μ*mol/l)	3.70 ± 0.32	3.59 ± 0.41	4.00 ± 0.27	0.66
GGT (IU/L)	37.37 ± 4.73	33.47 ± 17.64	34.01 ± 3.72	0.75
TG (mmol/l)	1.10 ± 0.12	1.88 ± 0.21	2.61 ± 0.52^a^	0.08
TC (mmol/l)	4.70 ± 0.59	5.02 ± 1.01	5.87 ± 1.58^a^	0.06
HDL-C (mmol/l)	1.20 ± 0.15	1.20 ± 0.28	1.23 ± 0.25	0.88
LDL-C (mmol/l)	3.07 ± 0.55	3.34 ± 0.78	3.88 ± 1.06^a^	0.01
Na^+^ (mmol/l)	139.54 ± 4.10	139.97 ± 3.94	139.51 ± 6.12	0.91
K^+^ (mmol/l)	3.89 ± 0.25	3.90 ± 0.18	3.27 ± 0.58^a^	<0.01
POP (mmol/l)	295.11 ± 7.77	302.83 ± 8.93	303.45 ± 12.32	0.21
BUN (mmol/l)	4.29 ± 0.98	5.20 ± 1.36	6.07 ± 2.42^a^	<0.01
eGFR (ml/min.1.73m^2^)	112.49 ± 13.61	117.31 ± 25.21^a^	164.72 ± 36.25^ab^	<0.01
sCr (*μ*mol/l)	58.97 ± 11.86	64.26 ± 14.15	64.19 ± 40.7	0.62
sUA (*μ*mol/l)	248.42 ± 56.95	303.31 ± 85.09^a^	302.42 ± 106.88^a^	0.05

ALB: albumin; ALT: alanine aminotransferase; AST: aspartate transaminase; BUN: blood urea nitrogen; DBIL: direct bilirubin; DK: diabetic ketosis; eGFR: estimated glomerular filtration rate; FPG: fasting plasma glucose; GGT: glutamyltransferase; HbA1c: glycated hemoglobin; HDL-C: high-density lipoprotein cholesterol; IBIL: indirect bilirubin; LDL-C: low-density lipoprotein cholesterol; NC: normal control; NDK: nondiabetic ketosis; POP: plasma osmotic pressure; SCr: serum creatinine; SUA: serum uric acid; TBIL: total bilirubin; TC: total cholesterol; TG: triglyceride; TP: total protein. Data are expressed as mean ± SD. ^a^Compared with NC, *P* < 0.05; ^b^compared with NDK, *P* < 0.05.

**Table 2:** FPG and urine ketone before and after ketosis control.

	Before ketosis control (*n* = 29)	After ketosis control (*n* = 29)	*P* value
FPG (mmol/l)	16.68 ± 4.88	11.12 ± 3.67	0.873
Urine ketone (+-++++)	2.83 ± 1.03	0.00 ± 0.00	<0.01

FPG: fasting plasma glucose. Data are expressed as mean ± SD.

## Data Availability

The data used to support the findings of this study are available from the corresponding author upon request.
